# Prioritising public health: a qualitative study of decision making to reduce health inequalities

**DOI:** 10.1186/1471-2458-11-821

**Published:** 2011-10-20

**Authors:** Lois C Orton, Ffion Lloyd-Williams, David C Taylor-Robinson, May Moonan, Martin O'Flaherty, Simon Capewell

**Affiliations:** 1 University of Liverpool; Public Health and Policy; Psychology, Health and Society; 2nd Floor Block B Waterhouse Buildings; Liverpool L69 3GL, UK

## Abstract

**Background:**

The public health system in England is currently facing dramatic change. Renewed attention has recently been paid to the best approaches for tackling the health inequalities which remain entrenched within British society and across the globe. In order to consider the opportunities and challenges facing the new public health system in England, we explored the current experiences of those involved in decision making to reduce health inequalities, taking cardiovascular disease (CVD) as a case study.

**Methods:**

We conducted an in-depth qualitative study employing 40 semi-structured interviews and three focus group discussions. Participants were public health policy makers and planners in CVD in the UK, including: Primary Care Trust and Local Authority staff (in various roles); General Practice commissioners; public health academics; consultant cardiologists; national guideline managers; members of guideline development groups, civil servants; and CVD third sector staff.

**Results:**

The short term target- and outcome-led culture of the NHS and the drive to achieve "more for less", combined with the need to address public demand for acute services often lead to investment in "downstream" public health intervention, rather than the "upstream" approaches that are most effective at reducing inequalities. Despite most public health decision makers wishing to redress this imbalance, they felt constrained due to difficulties in partnership working and the over-riding influence of other stakeholders in decision making processes. The proposed public health reforms in England present an opportunity for public health to move away from the medical paradigm of the NHS. However, they also reveal a reluctance of central government to contribute to shifting social norms.

**Conclusions:**

It is vital that the effectiveness and cost effectiveness of all new and existing policies and services affecting public health are measured in terms of their impact on the social determinants of health and health inequalities. Researchers have a vital role to play in providing the complex evidence required to compare different models of prevention and service delivery. Those working in public health must develop leadership to raise the profile of health inequalities as an issue that merits attention, resources and workforce capacity; and advocate for central government to play a key role in shifting social norms.

## Background

The public health system in England is currently facing dramatic change as a result of spending cuts and structural reforms outlined in the coalition government's Public Health White Paper "Healthy Lives, Healthy People" [1]; as well as their controversial and contested National Health Service (NHS) White Paper "Equity and Excellence: Liberating the NHS" [2]. These reforms signify a major shift in how public health services will be provided and delivered. Most significantly, the Public Health White Paper sees the creation of a new, integrated, national public health service "Public Health England" and local public health teams being removed from the NHS and returning to Local Authorities. The new Local Authorities will have increased responsibilities to coordinate overall health policy for a geographic area, joining together the work of local government, the NHS and the new National Public Health Service [3]. The coalition government states a commitment to addressing health inequalities, and the recent QIPP (Quality, Innovation, Productivity, Prevention) agenda aims to force (amongst other things) a focus on disease prevention and health and wellbeing promotion [4]. However, it remains to be seen how this will be achieved.

The most important determinants of health and health inequalities have been demonstrated as the wider "upstream" social determinants (those things which cause ill health and the causes of these causes) [5]. Despite this knowledge, inequalities remain difficult to shift. While average life expectancy has increased [6-9], inequalities have proved resistant to the many attempts, and considerable finances, invested in tackling them. As far back as 2000, despite the recently elected Labour government pledging to address health inequalities, concerns that the problem was not receiving the attention it was due led the House of Commons to launch an inquiry into public health. The results highlighted how resources were being channelled to "fix and mend" solutions rather than longer term preventive approaches and a lack of joined-up working to address public health issues [10]. However, it was not until publication of the independent Wanless report in 2002 [11] that the Government formulated a co-ordinated response to the problem. Wanless argued that everyone in society had a role to play in reducing inequalities, and that central government had a key responsibility to shift social norms, and enable individuals to make healthy choices, through legislative activity including taxes, subsidies, service provision, regulation and information [12]. His suggestions went against contemporary government thinking, which was largely targeted at addressing the "health gap" - ie, improving the health of the poorest, or those in the poorest areas, faster than the rest of the country, rather than tackling the broader social determinants [13]. Following publication of the report, the government invested in partnerships to tackle health inequalities. However, as Wanless observed in his 2004 evaluation [12], these efforts were quickly overshadowed by "lifestyle drift" with medicalised government policy tending to focus on providing advice and information encouraging individuals to take more responsibility for their own health, whilst ignoring the need to complement this with addressing the entrenched structural inequalities that exist in society.

By 2008, the UK government's Department of Health noted that despite the country as a whole being "healthier now than we have ever been" the health of the most disadvantaged had still "not improved as quickly as that of the better off" [14]. Their report went on to note that such differences were "often avoidable and always unjust". In the same year, the World Health Organization's Commission on Social Determinants of Health (CSDH) launched its report on reducing global health inequalities [15]. It contained three overarching recommendations: firstly, to improve daily living conditions; secondly to tackle the inequitable distribution of power, money, and resources; and thirdly to measure and understand the problem and assess the impact of action. In order to determine what England could learn from this report, the UK Department of Health asked Sir Michael Marmot to review the most effective evidence based approaches to reducing health inequalities in England from 2010. Marmot reinforced and extended the CSDH and Wanless' argument, suggesting that in order to influence socioeconomic factors, action is required by a wide range of policy makers including local government departments (not just those concerned primarily with health), as well as the third and private sector [16]. He argued that the social gradient in health must be tackled by universal action with a "scale and intensity that is proportionate to the level of disadvantage" (proportionate universalism [16]), rather than focusing solely on the most disadvantaged. Further, he criticised the dominant "lifestyles" focus, stating that to effectively impact on inequities in health, it is important not to blame individuals for their unhealthy behaviours but to create a society where the social and economic environment in which they live is conducive to good health [17].

With this renewed attention on the best approaches for tackling health inequalities both globally and in the UK, we felt it was particularly germain to explore the current experiences of those involved in decision making to reduce them, and to consider the opportunities and challenges facing the new public heath system in England. In order to shed light on these issues, we examined decision making for cardiovascular disease (CVD: the leading cause of death in the UK and worldwide [18]) as a case study.

## Methods

### Design

An in-depth qualitative design, employing interviews and focus group discussions and informed by ethnography, was chosen to allow exploration of the meanings and perceptions of participants against the backdrop of the overall context (culture) of decision making. The study was granted exemption from ethical review by the North West Research Ethics Committee in October 2009. It was conducted by a multidisciplinary team with varied backgrounds and experience, including: medical anthropology (LO: main researcher), clinical epidemiology (SC and MOF) and public health (DTR, FLW and MM).

### Participants and setting

Research participants were involved in decision making for CVD at a local, regional or national level in the UK. Potential participants were invited by email and written letter. They were provided with an information sheet and were encouraged to get in touch with the researcher if they wished to take part or if they had any questions. During early interviews, purposive sampling was used to seek as wide a spectrum of roles as possible. In later interviews we sought informants who would help in exploring the themes emerging from the analysis (theoretical sampling [19]). We planned to conduct between 30 and 40 interviews but kept interviewing until we had found what we needed to know in terms of addressing the research objectives [20]. Focus group discussions were conducted in order to further explore and test the main themes emerging from analysis of interview data. We aimed to recruit between six and 10 participants for each focus group based on the theoretical framework emerging from this analysis.

### Data generation

In-depth semi-structured interviews were the main method of data collection. After taking written informed consent, a topic guide was used as a prompt for questioning. The key consideration influencing the direction of each interview was the participant's answers to questions in terms of their individual experiences. The focus group discussions were conducted in order to explore key themes and some of the discrepancies and gaps in the interview data. After taking written informed consent, participants were presented with a summary of the interview findings and were encouraged to develop and reject the ideas presented to them, providing a method of respondent validation [21,22]. The first focus group took place on the site of a major decision making organisation; the second at a public health conference; and the third at a public health training event. All interviews and focus group discussions were recorded electronically and were transcribed verbatim. Detailed field notes were also taken in order to return context to the recorded material.

### Data analysis

Analysis occurred concurrently with data generation using the constant comparative method [19]. Transcripts and field notes were entered into the software package NVivo and were coded line-by-line based on the meanings, perspectives, and actions which they represented, and for contextual factors in their generation. A subset of 25 per cent of transcripts were double coded by two members of the research team, disagreements and insights were discussed and alternative interpretations were incorporated in the analysis [23]. The analysis was further tested during discussions with colleagues, through meetings of the project steering group, and in the focus group discussions. Through this process, the aim was to identify the "big ideas" (or themes) that were grounded in the data [24].

## Results

Seventy-nine public health decision makers in CVD from across the UK were approached to take part in an interview. Thirty-nine declined and 40 participated, including: seven CVD commissioners, four public health professionals, two data analysts, one researcher, and one knowledge manager from six NHS regions; two Local Authority (LA) staff; three staff with joint LA/NHS roles; one General Practice (GP) commissioner; seven public health academics; seven consultant cardiologists; one national guideline manager; one lay member of a guideline development group, one civil servant; and two CVD third sector staff. The first focus group included seven participants, all of whom had also taken part in an interview. They included: three consultant cardiologists; two public health consultants, a public health doctor, and a knowledge manager from one NHS region. The second focus group included 10 participants, all with an academic or practical interest in public health and the prevention of CVD. The third focus group included 20 participants involved in public health decision making in one region. Most interviews lasted about 45 minutes, ranging from 20 minutes to one hour and fifteen minutes. The first focus group lasted 70 minutes; and the second and third 60 minutes. The main findings from interviews and focus group discussions are presented together, below.

### The short term target- and outcome-led culture of the NHS

When this study was conducted, public health specialists were largely situated within the NHS (as a part of Primary Care Trusts), with some employed on joint contracts with local government. As such, they were absorbed into NHS culture. Whilst the government professed a commitment to tackling health inequalities, public health workers expressed concerns that the target- and outcome-led working environment created by current policy precluded a focus on addressing health inequalities. Public health specialists found themselves in a system in which the pressure to reduce waiting lists and meet budgetry demands often over-shadowed the need for preventive approaches.

*...acute and elective is where you break your targets, your waiting list targets so that has to be done and is what shouts the loudest*.

(public health specialist FGD3)

Public health specialists often felt unable to redress the balance away from the medical model and to divert the flow of investment from delivering services to those with an established condition to more "upstream" primary preventive approaches.

*There's national pressure, a national target, to reduce the all cause mortality by 2010. And that's a three year rolling average, so that gives us until December 2011. So we're having to take a fairly medical model with that and it's looking to see where we can have an impact fairly rapidly*.

(public health specialist 02)

There were two main reasons for this. Firstly, the government was seen to measure success in terms of short term impacts on health, rather than the long term solutions required to tackle entrenched inequalities in health.

*...the government is measuring us most of the time on short term things... And it just demonstrates the difficulty of having long term targets when there are lots of short term targets flying around*.

(public health consultant 02)

Secondly, there was an innate problem in measuring the effects of complex types of intervention necessary to reduce inequalities or even attributing cause and effect over long time periods. Some LA staff saw the task of disentangling the effects of public health interventions from other social and environmental factors as practically impossible.

*And it's very difficult to evaluate what difference is being made as well, that's the thing. Proving it is, um, is extremely difficult when there's so many other variables... so to do this work is [slight pause] almost a leap of faith, you know. We can, we can, we can carry on trying to prove this till we're blue in the face but, um, er, some of it is not provable as far as I can see*.

(local authority employee 01)

This problem was reflected in a lack of research evidence for the most cost-effective approaches to delivering population-level interventions to reduce inequalities.

*But it's also difficult to find the evidence as to what actually works, that is robust enough, because many of the things you're talking about I fully agree they're important. But being able to say that if we do this we would be able to have this level of impact is much easier to do for some of the treatments*.

(public health specialist FGD1)

Public health specialists felt that it was often impossible, or unethical, to generate this kind of evidence.

The limitations in everything we do is you just don't know enough and you sort of feel like you perhaps will never know enough because you need to formulate policy you really need intervention evidence, and you either don't have that, or it would be unethical to try and get it. So that makes the decision making process extremely difficult

(public health academic 03)

Without evidence, it was difficult to demonstrate the importance of investing resources in "upstream" approaches to public health (as opposed to "downstream" service development). In some regions PCT and LA staff were working together with academics to overcome this problem by developing novel approaches to measuring the health impacts of their initiatives.

*So we did a long drawn out process, lots of academic consensus and so on and produced guidance to the transport planners on how to value health better and then went a few steps further forward and produced a tool for cycling that means if someone is able to estimate how many cycle journeys a new project or indeed a new policy will create, and how long each journey will be, then they can produce a value for that*.

(public health academic 01)

However, most felt that there was still a long way to go to fully measure the impact of these approaches so that they could be valued in the same way as more "downstream" approaches.

### Balancing public demand for acute services

Another factor felt to contribute to the diversion of resources from "upstream" approaches was public pressure, expressed by local constituents, for expanded access to acute services. This was heightened by the recent squeeze on budgets.

*... if we have more influence over our own money that's fine, but you've got to balance that against public opinion and public want - the public want hospitals and they want expensive cancer drugs and all of that*.

(Joint LA/NHS employee 03)

You don't have people jumping up and down that we don't have enough smoking cessation programmes, you know?

(public health specialist 07)

Many decision makers felt that their attempts to reduce health inequalities were being systematically undervalued by this medicalised culture.

*I'd say the public health approaches are being undervalued, that's where the big savings are to be made and, um, I don't think we're investing enough in those. We're concentrating on treating the patient rather than preventing the patient being there in the first place*.

(joint LA/NHS employee 02)

They feared for the long term consequences for the population they were serving.

I think it's a very difficult situation for health really but there needs to be some sort of re- redress, you know, the balance has gone far too far, and the problem here is that you'll see these benefits many years down the line rather than on the health time frame which is very short. So, um, I don't know, it's, you know, complicated, there's no quick fixes there...

(LA employee 01)

### The importance of workforce capacity to address health inequalities

Some might say this under-valuing of long term "upstream" approaches to reducing inequalities reflects a failure on the part of public health professionals to raise the importance of the issue. In our study, most informants expressed a desire to push long term preventive measures. However, a wide range of different actors are involved in public health decision making processes. Those from different professional backgrounds worked within many different interpretations of how health inequalities might best be tackled. In particular, commissioners often had a very different perspective to those with a specialist public health background. In order to meet targets, some commissioners felt that focussing on the management of those with an established condition (secondary prevention) was the best way to reduce inequalities.

... we're trying to perhaps shift the balance slightly and put more into the, into the kind of prevention side and do a lot of services out in the community where patients have got management plans...

(CVD commissioner 02)

On the other hand, some commissioners prioritised primary prevention. However, in an approach which reflects the government's history of framing health inequalities as a problem based on a "health gap", they tended to prefer focussing on identifying and targeting interventions at those considered to be "high risk", "deprived" or "easy to miss" rather than adopting more effective population-wide approaches. These targeted initiatives were considered to have a more immediate and noticeable impact at a local level.

*So we feel that by going to certain populations in the real deprived areas for example that it's gonna have a bigger impact upon health inequalities because these people are much harder to reach, so to speak*.

(CVD commissioner 03)

Most public health professionals advocated for population-level approaches to reducing health inequalities. However, others showed a distinct deviation from what might be considered the traditional social or structuralism paradigm.

*I'm a bit hazy on primary prevention actually because most of the stuff I've done is on secondary prevention*.

(public health specialist 02)

This may reflect previous medical training or an enculturation into the medicalised NHS.

Another factor limiting the ability of public health decision makers to advocate for effective strategies to tackle health inequalities is a lack of capacity to interpret and apply the complex (and scant) research evidence.

*...you'll go up say to one of the lead commissioners I know upstairs and you say what are your needs and requirements over the next year in terms of research and they said straight away, oh research, oh it scares me*.

(CVD commissioner 02)

In the face of more convincing evidence for "downstream" interventions, this contributes to the difficulty in defending "upstream" approaches within the dominant evidence-based policy culture. Focus group informants discussed the importance of capacity building to increase the ability of decision makers to access and use complex public health research evidence. Some local programmes were underway with the specific purpose of supporting staff to include research evidence in their decision making processes. However, informants felt that understanding and using research should be an inherent and explicit part of training (or even a job requirement) for all those involved in public health decision making, and should start from the earliest possible stage. One suggestion for increasing the understanding of research was to introduce structures into the workplace that encourage and enable public health decision makers to negotiate the rigorous governance requirements and conduct their own research.

### Stakeholder power shapes decision making processes

As outlined above, there are many different interpretations of best way to address health inequalities. These interpretations are transferred, exchanged and adapted during decision making processes. Inevitably, the most powerful actor is likely to have the largest influence over the adopted interpretation. At the time of our study, NHS commissioners appeared to have most control in terms of decisions on investment of resources. As a result of the recent drive for efficiency savings, they felt compelled to take difficult decisions in distributing limited resources between primary prevention, prevention of recurrence or progression amongst those with existing disease (secondary prevention) and the immediate medical care of those with an established condition.

*...long term prevention, primary prevention, still has to run alongside, cause because otherwise we're not stopping the flow of people coming into the system. So you do still have to do the prevention, but it's not about just doing the prevention and letting this cohort trundle to a natural death; it's about doing the prevention plus at the same time doing the immediate finding people and managing them. Plus those people who are already at the end of their life, managing them more effectively so we spend less in hospital. [Loudly] We're having to do all of the layers all at the same time*.

(CVD commissioner 01)

Commissioners described feeling constrained to balance investment for preventative work whilst there was seen to be a large cohort in need of immediate medical intervention.

...*we all hesitate but investment in "upstream" preventative work, it pays dividends in the end, but you are always coping with the ones that haven't had the benefit from the prevention so you have still got to fund their care, and so what do you do*.

(CVD commissioner 04)

Furthermore, as their performance was assessed on the basis of meeting budgetry demands, their focus was often on avoiding costly hospitalisation, in the short term, amongst those with an established condition.

*...and we do some things to kind of prevent so many patients going into an acute phase of their illness and needing really high cost, high intensive treatment*.

(CVD commissioner 02)

On the other hand, public health professionals based in the NHS felt limited in their ability to address health inequalities due to the largely local or regional nature of their work. They often saw the power to introduce population-wide primary preventive approaches as lying at the national or international level.

*Trying to address the primary prevention agenda, it's obvious that a large part of the agenda has to be addressed at national or EU levels*.

(public health specialist 05)

Conversely, third sector employees saw their job precisely as influencing the national and international agenda from the bottom up. Some reported having had considerable influence on government decision making.

*We've moved as an organisation from being a kind of more an external organisation to moving away and working more internally with government... many of the things we say to the government they take on board*.

(third sector worker FGD3)

At a local level, those based in local government were seen to be best placed to address primary prevention.

*If you look at PCTs, the priority's largely around treatment. If you look at Local Authorities for them the bias is going to be on more generic interventions which are much more wrapped around prevention*.

(public health specialist 06)

Joint appointments between the NHS and LA were also felt to allow the profile of "upstream" approaches to be effectively raised within NHS structures.

### The importance of partnership working to address health inequalities

Joint appointments were often cited as an example of effective partnership working.

However, the complexity of decision making across sectors was felt by some to constrain the ability to address health inequalities.

*It's a nightmare, god with cardiovascular (laughs) well where do you stop? They look at government policy, national government, regional government, European government, you know economic policy, different types of political and social organisations so you know the breadth of it is immense*.

(public health academic 04)

In particular, there were cultural issues in working with a broad range of partners, such as a lack of shared values and language.

When you speak to local authority representatives, it's erm, it's like talking to an alien. And they feel the same to us because we use acronyms in the NHS like QUIPP and DOUGIE all that sort of stuff. So we're trying to get a foot in both camps really as a starter for six

(public health academic 05)

Furthermore, there were concerns that partner organisations (outside of the NHS) were not audited in terms of the same targets or outcomes, contributing to difficulties in demonstrating the impact of these wider approaches in reducing health inequalities.

### Implications for NHS and Public Health reforms in England

As our study has highlighted, one of the main barriers to addressing health inequalities is the medicalisation of the public health system and the over-riding influence of "downstream" targets and outcomes. With the proposed move of the public health function to local authorities in England there may be opportunities to break away from this medicalisation. However, there are also some important dangers in the proposals outlined in the recent White Papers. Reflecting the tendency for "lifestyle drift", there are plans to further shift decision making power to the local level, with a focus on encouraging individuals to take responsibility for their own health behaviours, rather than the government taking the lead in creating healthy environments. Public health professionals taking part in our study expressed concerns about these proposals.

*...at the moment there's a big push to devolve power to the local level... we need to make sure that you know at the centre there is still that capacity and opportunity for the Director of Public Health and others working in public health to address some of the determinants of health which need a national or international response*.

(third sector worker 04)

*...somehow the state was too involved in the past, when I saw it as barely involved at all and that these are matters of individual responsibility when individual responsibility is absolutely no safeguard against an ecological setting which is designed to overcome individual responsibility*.

(public health academic 02)

Some third sector staff saw their role as highlighting the inadequacy of the government's proposals for addressing the wider determinants of health.

*What PCT's and local organisations fail to do is to recognise publicly the importance of the national large scale complex interventions and to demand them and express support for them. Because it's only by getting demand from the periphery that we can put pressure on the centre to carry out those sorts of things*.

(third sector worker FGD3)

In order to ensure these issues are fully recognised, it is imperative that all those involved in decision making for public health bring their concerns to the attention of central government.

## Discussion

This qualitative study has revealed many tensions in investing in the "upstream" primary prevention initiatives that are most effective for reducing health inequalities. It describes the short term target- and outcome-led culture of the NHS, and the drive to achieve "more for less" as well as the need to address public demand for acute services; all of which lead to investment of the public health budget in treatment and the management of those who have already experienced a cardiovascular event (secondary prevention) rather than more "upstream" primary preventive approaches. Despite most public health decision makers wishing to redress the imbalance, they felt constrained in their ability to do this. Reasons included difficulties in partnership working and the over-riding influence of certain stakeholders in decision making processes (for whom there are often differing perceptions of the best approaches to addressing health inequalities as well as many competing pressures for the limited resources they control). Many decision makers felt that public health approaches were currently being systematically undervalued and were concerned about the consequences. This highlights the need for leadership in raising the profile of the health inequalities agenda, and the importance of developing workforce capacity to address it. There is a need to facilitate the use of the complex evidence for the effectiveness of public health interventions to better advocate for these approaches. In the light of the recent White Papers, this study also reveals the opportunities for public health in moving away from the medical paradigm of the NHS as well as the dangers in further neglecting action by central government to shift social norms.

For this study, a qualitative design, informed by ethnography, was adopted in order to probe in-depth into the experiences and perceptions of participants, against the backdrop of the overall context (culture) of decision making. The typical ethnographic approach, based at one site and involving extended participant observation, was modified for pragmatic and practical reasons. Firstly, we wanted to understand the process of decision making from the perspective of various different players in the process. Consequently, we explored decision making processes at a number of different sites. We used interviews and focus group discussions to gather data across these sites (rather than relying on observation.) Ethnographers often see the reactivity produced through such "solicited accounts" as a form of bias that precludes the perspectives of participants from emerging [21]. In order to minimise this reactivity, a brief topic guide and non-directive questioning were used during interviews and focus group discussions to enable participants to dictate the direction of the encounter and to allow for exploration of those topics of most relevance to them. Further, the process of analysis and interpretation adopted in this study was critical of the values, ideas and presumptions that the researcher brought to the research as a co-participant in each encounter.

Our findings, highlighting the struggle to prioritise public health, reinforce those from previous studies. Despite much having been written about health inequalities, so far there has been little effective action to reduce them. As Hunter [[25], p145] states "we have failed to put health before health care". In exploring the reasons for this, Blackman et al [26] describe the issue as a "wicked problem" for which there are no clear solutions. Those working in public health have been constrained by the "messiness" of the system, with its "fuzzy boundaries", changing membership and the lack of a clear idea of its core function and purpose [[25], p5]. There is uncertainty about the precise causes of health inequalities and the solutions, and a need to work in partnership across all sectors of society it order to tackle them. This has led to difficulties in knowing how to proceed, and a consequent sidelining of the health inequalities issue.

Our study and others have shown how the target- and outcome-led culture (introduced with New Labour in 1997) within which public health has been operating distorts priorities and has further contributed to the marginalisation of the inequalities agenda [11]. It leads practitioners to focus on what can be measured rather than what is important [26], with investment being diverted away from public health. Marks [27] and Blackman [28] argue that in order to address health inequalities, greater attention should be paid to the ways in which the inequalities agenda is embedded within local monitoring and decision making processes. Not only will this facilitate public health professionals in focussing more "upstream", it will also help recognise the contributions already being made to public health by LA activity (which participants in our study felt were currently being overlooked). As Wanless [11] warns, health inequalities targets must be practical and evidence based, rather than aspirational, or they will remain marginalised. This will not be easy. There is a lack of a strong evidence base for evaluations of wider public health interventions and in particular those policies which affect the social determinants of health and health inequalities [29], to guide action. There is an urgent need for empirical data on the cost effectiveness of different approaches to improving health and reducing inequalities. However, unlike for much of medicine, we will never be sure of what will work a priori. There will always be a need to tread lightly and to evaluate [28].

Hunter and Marks argue that the inability to see that public health is much wider than the NHS has also contributed to the marginalisation of action to reduce health inequalities [30]. All sectors involved in decision making for public health should be held accountable for the impacts of their policies on inequalities and the social determinants of health. The UK NHS and the public health function are both at a time of great flux. Our study adds to the health inequalities debate, by revealing the potential impact of the proposed changes to the public health system in England. The new reforms raise opportunities for public health to create a new identity for itself within LA and to manage its operations based on appropriate health inequality impacts. However, the background and training of public health staff, and the structures within which they are required to work, not simply where they are physically located, are also important factors in achieving this transition. Hunter [29] suggests that medical practitioners hold too much power in the current system. Senior positions continue to be dominated by those with a medical background. Under the new reforms they will soon be working in closer proximity with partners from a social and structuralist paradigm. Success in addressing health inequalities will depend on the forging of effective working relationships with these partners. Traditionally, local government has played a key role in public health through its work on sewers and sanitation, food hygiene and environmental health. Today it could play a key role in tackling the obesogenic environment we live in, and which is detrimental to our health, through the provision of cycle lanes, smoking measures, fast food planning, and other measures [31].

However, this action needs to be complemented with higher level policy to shift wider social norms. The government's plans to further devolve power to the local level demonstrates that the warnings of Wanless [11,14] and Marmot [16] have once again been ignored. Only when all sectors are held accountable for their contribution towards these targets can population-wide approaches to public health be suitably valued. The government must show leadership and enable local and regional decision makers to address inequalities.

## Conclusions

The new government proposes radical NHS reforms and the creation of a new public health function. In order for health inequalities to be effectively tackled, it is vital that the effectiveness and cost effectiveness of all new and existing policies and services affecting public health are measured in terms of their impact on the social determinants of health and health inequalities. Researchers will therefore have a vital role to play in providing the complex evidence required to compare different models of prevention and service delivery. Furthermore, those working in public health must develop leadership to raise the profile of the health inequalities issue ensuring it receives the resources, workforce capacity and attention it is due; and advocating for central government to play a key role in shifting social norms.

## Competing interests

The authors declare that they have no competing interests.

## Authors' contributions

LO developed the study materials, conducted interviews and focus group discussions, analysed the data and wrote the manuscript. FLW was an applicant for grant funding, assisted in the development of study materials and the analysis of the data, and commented on drafts. DTR assisted in the development of study materials and the analysis of the data, and commented on drafts. MM assisted in the analysis of the data and commented on drafts. MOF assisted in the development of study materials and commented on drafts. SC developed and managed the original project, obtained funding, and helped draft the first and final version of the manuscript. All authors read and approved the final manuscript.

## Pre-publication history

The pre-publication history for this paper can be accessed here:

http://www.biomedcentral.com/1471-2458/11/821/prepub
